# Crystal structure of (*E*)-1-{2-[(5,5-dimethyl-1,3,2-dioxaphosphinan-2-yl)­oxy]naphthalen-1-yl}-*N*-(4-fluoro­phen­yl)methanimine

**DOI:** 10.1107/S2056989014026838

**Published:** 2015-01-01

**Authors:** Musa A. Said, Bayan L. Al Belewi, David L. Hughes

**Affiliations:** a Chemistry Department, Taibah University, PO Box 30002, Code 14177, Al-Madinah Al-Munawarah, Kingdom of Saudi Arabia; b School of Chemistry, University of East Anglia, Norwich NR4 7TJ, England

**Keywords:** crystal structure, phosphites, 1,3,2-dioxaphosphinan-2-oxy, naphthalene, C—H⋯π inter­actions

## Abstract

In the title compound, C_22_H_21_FNO_3_P, a 1,3,2-dioxaphosphinan-2-yloxy derivative, three O atoms are bonded in a trigonal–pyramidal manner to the P atom. The exocyclic P—O bond is significantly longer than the two endocyclic P—O bonds, *viz.* 1.6678 (12) Å compared to 1.6046 (13) and 1.6096 (12) Å. The six-membered ring which includes the P atom has a chair conformation. The fluoro­phenyl ring is inclined to the naphthalene ring system by 24.42 (7)°. In the crystal, mol­ecules are linked *via* C—H⋯π inter­actions, forming slabs lying parallel to (10-1).

## Abstract 2

In the title compound, the six-membered ring which includes the P atom has a chair conformation and three O atoms are bonded in a trigonal–pyramidal manner to the P atom. In the crystal, mol­ecules are linked *via* C—H⋯π inter­actions, forming slabs lying parallel to (10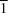).

## Chemical context   

Many phospho­rus and/or nitro­gen based ligands bind strongly to transition metals and they offer a wide range of properties and basicities due to the large variety of accessible substituents (Crabtree, 2005 ▸; Joslin *et al.*, 2012 ▸; Kuehl, 2005 ▸; Tolman, 1977 ▸). The title compound is an example of a phospho­rus-nitro­gen bidentate ligand. Complexation experiments with such ligands could result in the isolation of mono- or bi-nuclear complexes (van den Beuken *et al.*, 1997 ▸). Examples of bidentate ligands with phospho­rus and nitro­gen donor groups bonded to transition metals have been shown to be effective cross-coupling catalysts (Hayashi & Kumada, 1985 ▸). The present work is a continuation of the investigation into the synthesis and study of more bi- and tri-cyclic, penta- and hexa-­coordinated phospho­ranes to form anionic, neutral and zwitterionic compounds (Said *et al.* 1996 ▸; Timosheva *et al.* 2006 ▸; Kumara Swamy & Kumar, 2006 ▸).
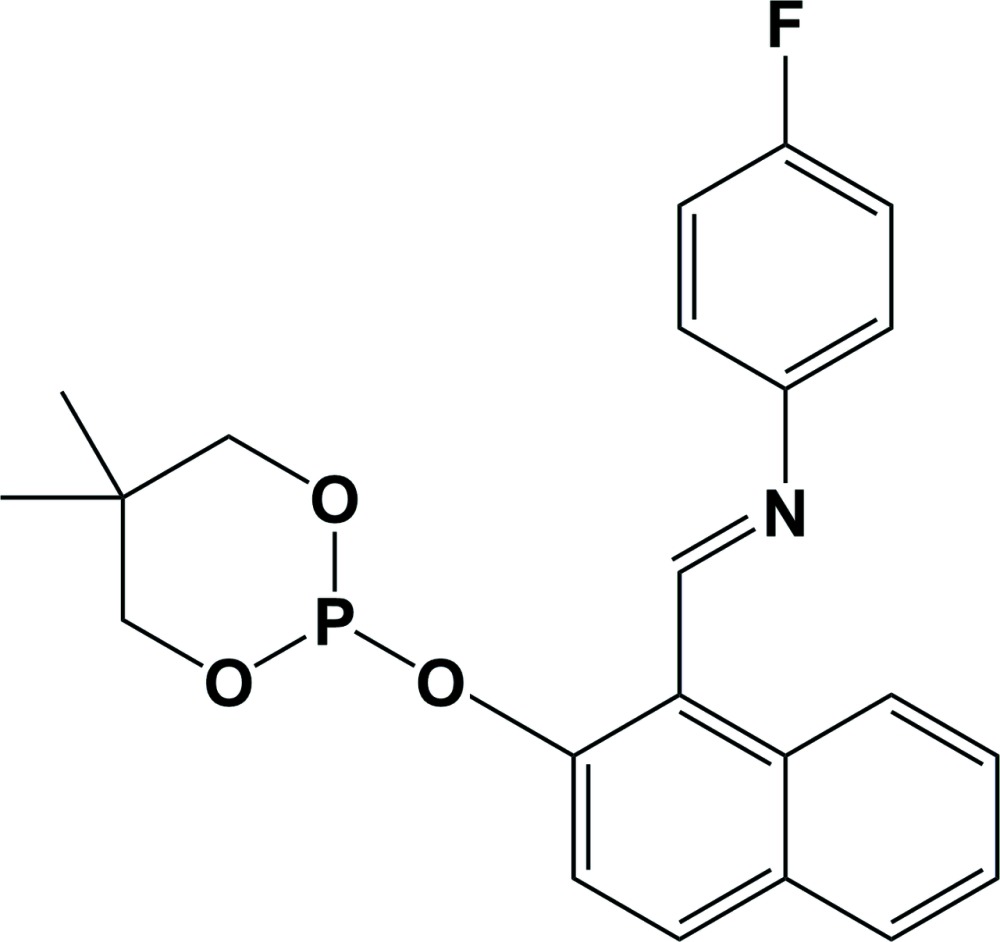


## Structural commentary   

**Figure 1:**
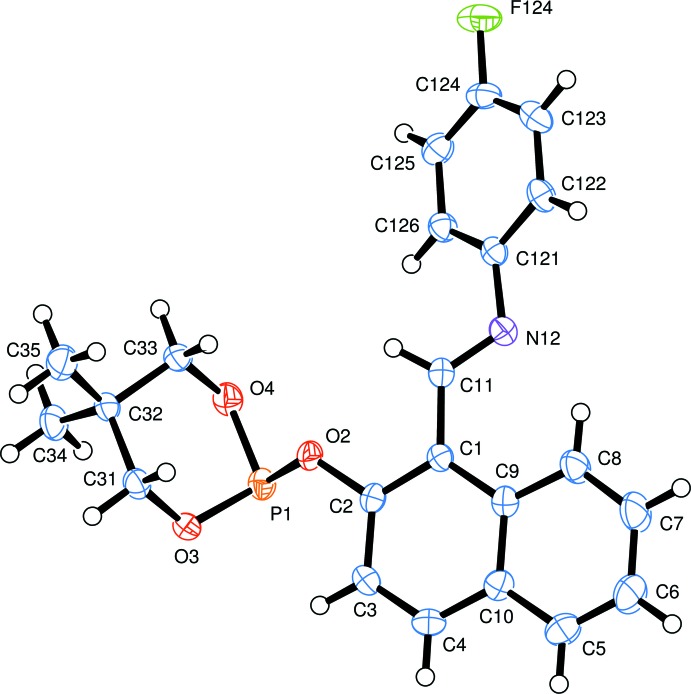
The mol­ecular structure of the title compound, showing the atom labelling. Displacement ellipsoids are drawn at the 50% probability level.

The mol­ecular structure of the title compound, Fig. 1 ▸, shows that the three oxygen atoms about the phospho­rus atom are bonded in a trigonal pyramidal form. The O—P—O angles are in the range of 96.35 (6) to 102.37 (6)°. The P1—O2 bond length [1.6678 (12) Å] is significantly longer than the other P—O bonds [1.6046 (13) and 1.6096 (12) Å]. The six-membered ring which includes the phospho­rus atom has a chair conformation. The fluoro­phenyl ring is inclined to the naphthalene ring system by 24.42 (7)°. The mol­ecule has an *E* conformation about the C=N bond (Fig. 1 ▸).

## Supra­molecular features   

**Figure 2:**
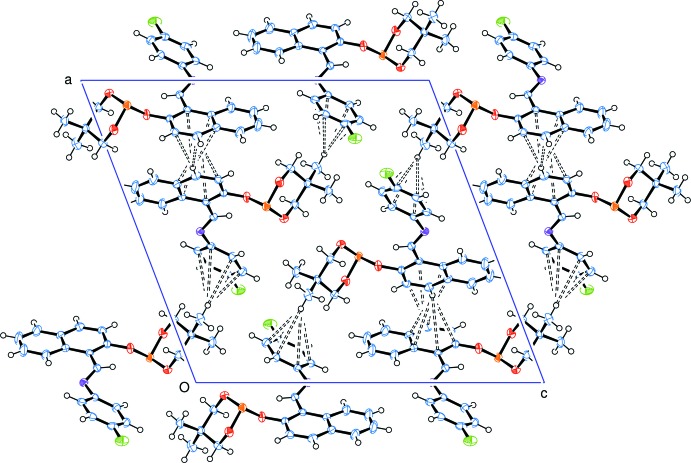
A view along the *b* axis of the crystal packing of the title compound showing the H⋯C contacts (dashed lines) of the C—H⋯π weak interactions (see Table 1 ▸ for details).

In the crystal, mol­ecules are linked *via* C—H⋯π inter­actions (Table 1 ▸), forming slabs lying parallel to (10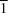), as shown in Fig. 2 ▸.

## Synthesis and crystallization   

To 1.02 g (6.05 mmol) of 2-chloro-5,5-dimethyl-1,2,3-dioxaphosphinane in 40 ml of dry di­chloro­methane was added 1.61 g (6.05 mmol) of (*E*)-1-[(4-fluoro­phenyl­imino)­meth­yl]­naphthalene-2-ol in 10 ml of dry di­chloro­methane. The mixture was refluxed under a slow flow of nitro­gen for 4 h. The solvent was reduced to 5 ml under vacuum and 3 ml of dry *n*-hexane were added to afford the title compound as a pale-yellow crystalline solid (yield 2.07 g, 86%; m.p. 401–405 K). ^1^H NMR (CDCl_3_, 450 MHz): δ 9.16 (*s*, 1H, CHN), 7.83–7.01 (*m*, 10H, Ar—H), 4.22 (*d*, 2H, CH_2_), 3.40 (*t*, 2H, CH_2_), 1.23 (*s*, 3H, CH_3_), 0.65 (*s*, 3H, CH_3_). ^13^C NMR (CDCl_3_, 450 MHz): δ 162.46–115.62 (aromatic carbons), 69.86 (1C, CMe_2_), 32.95 (2C, CH_2_), 22.46 (2C, CH_3_). ^31^P NMR (CDCl_3_, 450 MHz): δ 116.31. ^19^F NMR (CDCl_3_, 450 MHz): δ −116.10.

## Refinement   

Crystal data, data collection and structure refinement details are summarized in Table 2 ▸. The H atoms were included in idealized positions and treated as riding atoms: C—H = 0.93–0.97 Å with *U*_iso_(H) = 1.5*U*_eq_(C) for methyl H atoms and = 1.2*U*_eq_(C) for other H atoms.

## Supplementary Material

Crystal structure: contains datablock(s) I. DOI: 10.1107/S2056989014026838/su5030sup1.cif

Structure factors: contains datablock(s) I. DOI: 10.1107/S2056989014026838/su5030Isup2.hkl

Supporting information file. DOI: 10.1107/S2056989014026838/su5030Isup3.cml

CCDC reference: 1037929

Additional supporting information:  crystallographic information; 3D view; checkCIF report

## Figures and Tables

**Table 1:** CH interactions (, ) *Cg*1 and *Cg*2 are the centroids of rings C1C4/C9/C10 and C121C126, respectively.

*D*H*A*	*D*H	H*A*	*D* *A*	*D*H*A*
C4H4*Cg*1^i^	0.93	2.70	3.456(2)	140
C35H35*C* *Cg*2^ii^	0.96	2.94	3.878(2)	167

Symmetry codes: (i) 
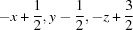
; (ii) 
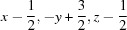
.

**Table 2:** Experimental details

Crystal data	
Chemical formula	C_22_H_21_FNO_3_P
*M*_r_	397.37
Crystal system, space group	Monoclinic, *P*2_1_/*n*
Temperature (K)	140
*a*, *b*, *c* ()	18.3667(8), 5.7898(2), 19.7710(7)
()	110.870(4)
*V* (^3^)	1964.50(13)
*Z*	4
Radiation type	Mo *K*
(mm^1^)	0.17
Crystal size (mm)	0.40 0.11 0.07

Data collection	
Diffractometer	Oxford Diffraction Xcalibur 3/Sapphire3 CCD
Absorption correction	Multi-scan (*CrysAlis PRO*; Oxford Diffraction, 2010 ▸)
*T*_min_, *T*_max_	0.790, 1.000
No. of measured, independent and observed [*I* > 2(*I*)] reflections	32284, 4518, 3624
*R*_int_	0.054
(sin /)_max_ (^1^)	0.650

Refinement	
*R*[*F* ^2^ > 2(*F* ^2^)], *wR*(*F* ^2^), *S*	0.044, 0.097, 1.05
No. of reflections	4518
No. of parameters	253
H-atom treatment	H-atom parameters constrained
_max_, _min_ (e ^3^)	0.26, 0.34

Computer programs: *CrysAlis PRO* (Oxford Diffraction, 2010 ▸), *SHELXS97*, *SHELXL97* and *SHELXL2014* (Sheldrick, 2008 ▸), *ORTEPII* (Johnson, 1976 ▸) and *WinGX* (Farrugia, 2012 ▸).

## supplementary crystallographic information

### Crystal data

**Table:**

C_22_H_21_FNO_3_P	*F*(000) = 832
*M**_r_* = 397.37	*D*_x_ = 1.344 Mg m^−^^3^
Monoclinic, *P*2_1_/*n*	Mo *K*α radiation, λ = 0.71073 Å
*a* = 18.3667 (8) Å	Cell parameters from 5215 reflections
*b* = 5.7898 (2) Å	θ = 3.1–32.5°
*c* = 19.7710 (7) Å	µ = 0.17 mm^−^^1^
β = 110.870 (4)°	*T* = 140 K
*V* = 1964.50 (13) Å^3^	Prism, pale yellow
*Z* = 4	0.40 × 0.11 × 0.07 mm

### Data collection

**Table:**

Oxford Diffraction Xcalibur 3/Sapphire3 CCD diffractometer	4518 independent reflections
Radiation source: Enhance (Mo) X-ray Source	3624 reflections with *I* > 2σ(*I*)
Graphite monochromator	*R*_int_ = 0.054
Detector resolution: 16.0050 pixels mm^-1^	θ_max_ = 27.5°, θ_min_ = 3.1°
Thin–slice φ and ω scans	*h* = −23→23
Absorption correction: multi-scan (*CrysAlis PRO*; Oxford Diffraction, 2010)	*k* = −7→7
*T*_min_ = 0.790, *T*_max_ = 1.000	*l* = −25→25
32284 measured reflections	

### Refinement

**Table:**

Refinement on *F*^2^	Primary atom site location: structure-invariant direct methods
Least-squares matrix: full	Secondary atom site location: difference Fourier map
*R*[*F*^2^ > 2σ(*F*^2^)] = 0.044	Hydrogen site location: inferred from neighbouring sites
*wR*(*F*^2^) = 0.097	H-atom parameters constrained
*S* = 1.05	*w* = 1/[σ^2^(*F*_o_^2^) + (0.0347*P*)^2^ + 0.8268*P*] where *P* = (*F*_o_^2^ + 2*F*_c_^2^)/3
4518 reflections	(Δ/σ)_max_ < 0.001
253 parameters	Δρ_max_ = 0.26 e Å^−^^3^
0 restraints	Δρ_min_ = −0.34 e Å^−^^3^

### Special details

**Table:**

Geometry. All e.s.d.'s (except the e.s.d. in the dihedral angle between two l.s. planes) are estimated using the full covariance matrix. The cell e.s.d.'s are taken into account individually in the estimation of e.s.d.'s in distances, angles and torsion angles; correlations between e.s.d.'s in cell parameters are only used when they are defined by crystal symmetry. An approximate (isotropic) treatment of cell e.s.d.'s is used for estimating e.s.d.'s involving l.s. planes.
Refinement. Reflections were merged by *SHELXL* according to the crystal class for the calculation of statistics and refinement._reflns_Friedel_fraction is defined as the number of unique Friedel pairs measured divided by the number that would be possible theoretically, ignoring centric projections and systematic absences.

### Fractional atomic coordinates and isotropic or equivalent isotropic displacement parameters (Å^2^)

**Table:**

	*x*	*y*	*z*	*U*_iso_*/*U*_eq_	
C1	0.40211 (9)	0.4807 (3)	0.77326 (8)	0.0211 (3)	
C2	0.37237 (9)	0.3394 (3)	0.71306 (8)	0.0219 (3)	
C3	0.32866 (10)	0.1394 (3)	0.71261 (9)	0.0250 (4)	
H3	0.3085	0.0508	0.6708	0.030*	
C4	0.31611 (10)	0.0769 (3)	0.77383 (9)	0.0275 (4)	
H4	0.2892	−0.0588	0.7742	0.033*	
C5	0.32844 (12)	0.1500 (4)	0.89990 (10)	0.0382 (5)	
H5	0.3014	0.0142	0.8999	0.046*	
C6	0.35310 (13)	0.2835 (4)	0.96045 (11)	0.0494 (6)	
H6	0.3437	0.2381	1.0017	0.059*	
C7	0.39270 (13)	0.4898 (4)	0.96012 (11)	0.0480 (6)	
H7	0.4084	0.5826	1.0012	0.058*	
C8	0.40887 (11)	0.5581 (4)	0.90065 (9)	0.0349 (4)	
H8	0.4354	0.6958	0.9020	0.042*	
C9	0.38558 (9)	0.4212 (3)	0.83716 (9)	0.0240 (4)	
C10	0.34324 (10)	0.2143 (3)	0.83686 (9)	0.0262 (4)	
C11	0.45172 (9)	0.6736 (3)	0.76817 (8)	0.0221 (3)	
H11	0.4477	0.7220	0.7221	0.027*	
N12	0.49987 (8)	0.7800 (2)	0.82205 (7)	0.0240 (3)	
C121	0.54836 (9)	0.9486 (3)	0.80773 (9)	0.0228 (3)	
C122	0.56436 (10)	1.1496 (3)	0.84926 (9)	0.0269 (4)	
H122	0.5429	1.1695	0.8850	0.032*	
C123	0.61157 (10)	1.3197 (3)	0.83817 (10)	0.0304 (4)	
H123	0.6209	1.4555	0.8650	0.036*	
C124	0.64446 (10)	1.2835 (3)	0.78647 (10)	0.0300 (4)	
F124	0.69271 (6)	1.44882 (19)	0.77655 (7)	0.0435 (3)	
C125	0.63219 (10)	1.0855 (3)	0.74575 (10)	0.0301 (4)	
H125	0.6560	1.0644	0.7119	0.036*	
C126	0.58359 (10)	0.9183 (3)	0.75626 (9)	0.0260 (4)	
H126	0.5742	0.7839	0.7287	0.031*	
P1	0.41707 (3)	0.20397 (8)	0.60526 (2)	0.02624 (12)	
O2	0.38648 (7)	0.3996 (2)	0.65108 (6)	0.0262 (3)	
O3	0.33653 (7)	0.14447 (19)	0.54070 (6)	0.0269 (3)	
O4	0.45712 (7)	0.3839 (2)	0.56752 (6)	0.0293 (3)	
C31	0.29181 (10)	0.3271 (3)	0.49448 (9)	0.0270 (4)	
H31A	0.2717	0.4289	0.5227	0.032*	
H31B	0.2477	0.2601	0.4563	0.032*	
C32	0.34018 (10)	0.4681 (3)	0.46065 (8)	0.0247 (4)	
C33	0.41052 (10)	0.5660 (3)	0.52151 (9)	0.0272 (4)	
H33A	0.4426	0.6532	0.5008	0.033*	
H33B	0.3927	0.6710	0.5506	0.033*	
C34	0.36634 (11)	0.3186 (3)	0.40960 (9)	0.0338 (4)	
H34A	0.3974	0.1925	0.4362	0.051*	
H34B	0.3966	0.4105	0.3889	0.051*	
H34C	0.3214	0.2591	0.3717	0.051*	
C35	0.29039 (12)	0.6694 (3)	0.41909 (10)	0.0378 (5)	
H35A	0.3199	0.7601	0.3973	0.057*	
H35B	0.2754	0.7641	0.4518	0.057*	
H35C	0.2446	0.6105	0.3820	0.057*	

### Atomic displacement parameters (Å^2^)

**Table:**

	*U*^11^	*U*^22^	*U*^33^	*U*^12^	*U*^13^	*U*^23^
C1	0.0179 (8)	0.0223 (8)	0.0214 (8)	0.0023 (6)	0.0050 (6)	0.0006 (6)
C2	0.0218 (8)	0.0245 (9)	0.0197 (8)	0.0044 (6)	0.0078 (7)	0.0017 (6)
C3	0.0235 (9)	0.0243 (9)	0.0248 (8)	0.0000 (7)	0.0058 (7)	−0.0044 (7)
C4	0.0251 (9)	0.0240 (9)	0.0352 (9)	−0.0020 (7)	0.0131 (8)	0.0003 (7)
C5	0.0400 (11)	0.0440 (12)	0.0377 (10)	−0.0083 (9)	0.0223 (9)	0.0023 (9)
C6	0.0566 (14)	0.0693 (15)	0.0318 (10)	−0.0176 (12)	0.0274 (10)	−0.0035 (10)
C7	0.0543 (14)	0.0670 (15)	0.0287 (10)	−0.0202 (11)	0.0222 (10)	−0.0149 (10)
C8	0.0366 (10)	0.0438 (11)	0.0280 (9)	−0.0115 (9)	0.0159 (8)	−0.0094 (8)
C9	0.0196 (8)	0.0301 (9)	0.0223 (8)	0.0024 (7)	0.0074 (7)	−0.0004 (7)
C10	0.0226 (8)	0.0306 (9)	0.0271 (8)	0.0016 (7)	0.0107 (7)	0.0017 (7)
C11	0.0224 (8)	0.0232 (8)	0.0204 (8)	0.0037 (6)	0.0071 (7)	−0.0005 (6)
N12	0.0220 (7)	0.0263 (7)	0.0241 (7)	−0.0008 (6)	0.0088 (6)	−0.0031 (6)
C121	0.0196 (8)	0.0233 (8)	0.0228 (8)	0.0026 (6)	0.0043 (7)	−0.0005 (7)
C122	0.0204 (8)	0.0308 (10)	0.0266 (9)	0.0023 (7)	0.0048 (7)	−0.0056 (7)
C123	0.0227 (9)	0.0238 (9)	0.0373 (10)	0.0027 (7)	0.0016 (8)	−0.0044 (8)
C124	0.0210 (9)	0.0243 (9)	0.0395 (10)	−0.0016 (7)	0.0042 (8)	0.0083 (8)
F124	0.0348 (6)	0.0318 (6)	0.0615 (8)	−0.0075 (5)	0.0142 (6)	0.0086 (5)
C125	0.0288 (9)	0.0329 (10)	0.0305 (9)	0.0018 (8)	0.0129 (8)	0.0040 (8)
C126	0.0280 (9)	0.0239 (9)	0.0255 (8)	0.0009 (7)	0.0089 (7)	−0.0023 (7)
P1	0.0281 (2)	0.0278 (2)	0.0229 (2)	0.00239 (19)	0.00914 (18)	−0.00166 (18)
O2	0.0367 (7)	0.0246 (6)	0.0189 (6)	−0.0019 (5)	0.0118 (5)	−0.0021 (5)
O3	0.0337 (7)	0.0223 (6)	0.0239 (6)	−0.0050 (5)	0.0092 (5)	−0.0029 (5)
O4	0.0225 (6)	0.0387 (7)	0.0270 (6)	−0.0026 (5)	0.0092 (5)	−0.0019 (5)
C31	0.0238 (9)	0.0315 (10)	0.0237 (8)	−0.0029 (7)	0.0059 (7)	−0.0029 (7)
C32	0.0289 (9)	0.0258 (9)	0.0202 (8)	−0.0040 (7)	0.0096 (7)	−0.0042 (7)
C33	0.0311 (9)	0.0284 (9)	0.0245 (8)	−0.0076 (7)	0.0127 (7)	−0.0031 (7)
C34	0.0400 (11)	0.0401 (11)	0.0238 (9)	−0.0042 (9)	0.0143 (8)	−0.0083 (8)
C35	0.0477 (12)	0.0330 (11)	0.0294 (9)	0.0014 (9)	0.0096 (9)	0.0016 (8)

### Geometric parameters (Å, º)

**Table:**

C1—C2	1.386 (2)	C123—C124	1.376 (3)
C1—C9	1.442 (2)	C123—H123	0.9300
C1—C11	1.467 (2)	C124—F124	1.365 (2)
C2—O2	1.3843 (19)	C124—C125	1.372 (3)
C2—C3	1.407 (2)	C125—C126	1.382 (2)
C3—C4	1.359 (2)	C125—H125	0.9300
C3—H3	0.9300	C126—H126	0.9300
C4—C10	1.411 (2)	P1—O4	1.6046 (13)
C4—H4	0.9300	P1—O3	1.6096 (12)
C5—C6	1.360 (3)	P1—O2	1.6678 (12)
C5—C10	1.417 (2)	O3—C31	1.447 (2)
C5—H5	0.9300	O4—C33	1.455 (2)
C6—C7	1.399 (3)	C31—C32	1.525 (2)
C6—H6	0.9300	C31—H31A	0.9700
C7—C8	1.370 (3)	C31—H31B	0.9700
C7—H7	0.9300	C32—C33	1.527 (2)
C8—C9	1.416 (2)	C32—C35	1.528 (2)
C8—H8	0.9300	C32—C34	1.531 (2)
C9—C10	1.427 (2)	C33—H33A	0.9700
C11—N12	1.275 (2)	C33—H33B	0.9700
C11—H11	0.9300	C34—H34A	0.9600
N12—C121	1.416 (2)	C34—H34B	0.9600
C121—C122	1.394 (2)	C34—H34C	0.9600
C121—C126	1.398 (2)	C35—H35A	0.9600
C122—C123	1.380 (2)	C35—H35B	0.9600
C122—H122	0.9300	C35—H35C	0.9600
			
C2—C1—C9	118.02 (15)	F124—C124—C123	118.63 (16)
C2—C1—C11	117.04 (14)	C125—C124—C123	122.63 (17)
C9—C1—C11	124.85 (14)	C124—C125—C126	118.48 (17)
O2—C2—C1	118.08 (14)	C124—C125—H125	120.8
O2—C2—C3	119.20 (14)	C126—C125—H125	120.8
C1—C2—C3	122.71 (15)	C125—C126—C121	120.85 (16)
C4—C3—C2	119.39 (16)	C125—C126—H126	119.6
C4—C3—H3	120.3	C121—C126—H126	119.6
C2—C3—H3	120.3	O4—P1—O3	102.37 (6)
C3—C4—C10	121.18 (16)	O4—P1—O2	96.35 (6)
C3—C4—H4	119.4	O3—P1—O2	100.59 (6)
C10—C4—H4	119.4	C2—O2—P1	121.02 (10)
C6—C5—C10	121.11 (18)	C31—O3—P1	119.93 (10)
C6—C5—H5	119.4	C33—O4—P1	119.75 (10)
C10—C5—H5	119.4	O3—C31—C32	112.32 (13)
C5—C6—C7	119.46 (18)	O3—C31—H31A	109.1
C5—C6—H6	120.3	C32—C31—H31A	109.1
C7—C6—H6	120.3	O3—C31—H31B	109.1
C8—C7—C6	121.49 (19)	C32—C31—H31B	109.1
C8—C7—H7	119.3	H31A—C31—H31B	107.9
C6—C7—H7	119.3	C31—C32—C33	108.34 (13)
C7—C8—C9	120.68 (18)	C31—C32—C35	108.27 (14)
C7—C8—H8	119.7	C33—C32—C35	108.46 (14)
C9—C8—H8	119.7	C31—C32—C34	110.82 (14)
C8—C9—C10	117.75 (15)	C33—C32—C34	110.69 (14)
C8—C9—C1	123.49 (16)	C35—C32—C34	110.18 (14)
C10—C9—C1	118.76 (15)	O4—C33—C32	111.58 (13)
C4—C10—C5	120.70 (17)	O4—C33—H33A	109.3
C4—C10—C9	119.82 (15)	C32—C33—H33A	109.3
C5—C10—C9	119.48 (16)	O4—C33—H33B	109.3
N12—C11—C1	124.98 (15)	C32—C33—H33B	109.3
N12—C11—H11	117.5	H33A—C33—H33B	108.0
C1—C11—H11	117.5	C32—C34—H34A	109.5
C11—N12—C121	117.67 (14)	C32—C34—H34B	109.5
C122—C121—C126	118.57 (16)	H34A—C34—H34B	109.5
C122—C121—N12	118.25 (15)	C32—C34—H34C	109.5
C126—C121—N12	123.11 (15)	H34A—C34—H34C	109.5
C123—C122—C121	120.98 (17)	H34B—C34—H34C	109.5
C123—C122—H122	119.5	C32—C35—H35A	109.5
C121—C122—H122	119.5	C32—C35—H35B	109.5
C124—C123—C122	118.44 (16)	H35A—C35—H35B	109.5
C124—C123—H123	120.8	C32—C35—H35C	109.5
C122—C123—H123	120.8	H35A—C35—H35C	109.5
F124—C124—C125	118.72 (17)	H35B—C35—H35C	109.5
			
C9—C1—C2—O2	178.35 (14)	C11—N12—C121—C126	−41.1 (2)
C11—C1—C2—O2	−4.8 (2)	C126—C121—C122—C123	2.4 (2)
C9—C1—C2—C3	−1.5 (2)	N12—C121—C122—C123	179.62 (15)
C11—C1—C2—C3	175.39 (14)	C121—C122—C123—C124	−1.9 (2)
O2—C2—C3—C4	178.58 (15)	C122—C123—C124—F124	−178.44 (15)
C1—C2—C3—C4	−1.6 (2)	C122—C123—C124—C125	0.0 (3)
C2—C3—C4—C10	2.8 (3)	F124—C124—C125—C126	179.69 (15)
C10—C5—C6—C7	1.0 (3)	C123—C124—C125—C126	1.3 (3)
C5—C6—C7—C8	−1.5 (4)	C124—C125—C126—C121	−0.7 (3)
C6—C7—C8—C9	0.2 (3)	C122—C121—C126—C125	−1.1 (2)
C7—C8—C9—C10	1.6 (3)	N12—C121—C126—C125	−178.18 (15)
C7—C8—C9—C1	−178.70 (19)	C1—C2—O2—P1	133.32 (13)
C2—C1—C9—C8	−176.40 (16)	C3—C2—O2—P1	−46.84 (19)
C11—C1—C9—C8	7.0 (3)	O4—P1—O2—C2	−156.24 (12)
C2—C1—C9—C10	3.3 (2)	O3—P1—O2—C2	99.87 (12)
C11—C1—C9—C10	−173.29 (15)	O4—P1—O3—C31	−42.49 (13)
C3—C4—C10—C5	178.69 (17)	O2—P1—O3—C31	56.49 (12)
C3—C4—C10—C9	−0.9 (3)	O3—P1—O4—C33	43.33 (12)
C6—C5—C10—C4	−178.77 (19)	O2—P1—O4—C33	−59.01 (12)
C6—C5—C10—C9	0.8 (3)	P1—O3—C31—C32	54.06 (17)
C8—C9—C10—C4	177.50 (16)	O3—C31—C32—C33	−57.15 (18)
C1—C9—C10—C4	−2.2 (2)	O3—C31—C32—C35	−174.59 (13)
C8—C9—C10—C5	−2.1 (2)	O3—C31—C32—C34	64.47 (18)
C1—C9—C10—C5	178.22 (16)	P1—O4—C33—C32	−55.56 (16)
C2—C1—C11—N12	−160.05 (16)	C31—C32—C33—O4	57.62 (18)
C9—C1—C11—N12	16.6 (3)	C35—C32—C33—O4	174.93 (14)
C1—C11—N12—C121	174.38 (14)	C34—C32—C33—O4	−64.08 (18)
C11—N12—C121—C122	141.88 (16)		

### Hydrogen-bond geometry (Å, º)

Cg1 and Cg2 are the centroids of rings C1–C4/C9/C10 and C121–C126, respectively.

**Table:**

*D*—H···*A*	*D*—H	H···*A*	*D*···*A*	*D*—H···*A*
C4—H4···*Cg*1^i^	0.93	2.70	3.456 (2)	140
C35—H35*C*···*Cg*2^ii^	0.96	2.94	3.878 (2)	167

Symmetry codes: (i) −*x*+1/2, *y*−1/2, −*z*+3/2; (ii) *x*−1/2, −*y*+3/2, *z*−1/2.
